# Mapping nationally and globally at-risk species to identify hotspots for (and gaps in) conservation

**DOI:** 10.1098/rspb.2022.2307

**Published:** 2023-03-29

**Authors:** Marie E. Hardouin, Anna L. Hargreaves

**Affiliations:** Department of Biology, McGill University, 1205 Dr. Penfield Ave, Montreal, Quebec Canada, H3A 1B1

**Keywords:** species at risk, protected areas, conservation hotspots, peripheral species, Canada

## Abstract

Protecting habitat of species at risk is critical to their recovery, but can be contentious. For example, protecting species that are locally imperilled but globally common is often thought to distract from protecting globally imperilled species. However, such perceived trade-offs are based on the assumption that threatened groups have little spatial overlap, which is rarely quantified. We compiled range maps of terrestrial species at risk in Canada to assess the geographic overlap of nationally and globally at-risk species with each other, among taxonomic groups, and with protected areas. While many nationally at-risk taxa only occur in Canada at their northern range edge, they are not significantly more peripheral in Canada than globally at-risk species. Further, 56% of hotspots of nationally at-risk taxa are also hotspots of globally at-risk species, undercutting the perceived trade-off in their protection. While strong spatial overlap across threat levels and taxa should facilitate efficient habitat protection, less than 7% of the area in Canada's at-risk hotspots is protected, and two-thirds of nationally and globally at-risk species in Canada have less than 10% of their Canadian range protected. Our results counter the perception that protecting nationally versus globally at-risk species are at odds, and identify critical areas to target as Canada strives to increase its protected areas and promote recovery of species at risk.

## 1. Introduction

Habitat destruction is one of the primary threats to global biodiversity [1]. Indeed, the loss and degradation of habitat is the main threat to 85% of species listed in the International Union for the Conservation of Nature (IUCN) Red List of Threatened Species [2] and a leading barrier to the recovery of imperilled species [3,4]. Protecting habitat through the establishment of protected areas is thus one crucial component of species-at-risk conservation. At the local scale, protected areas can alleviate land-conversion pressures on existing populations of species at risk, and at the landscape scale they can provide crucial habitat corridors that could facilitate species range shifts in response to climate change [5,6].

While publicly protected areas are a key conservation tool in the global fight to protect biodiversity, it can be challenging to establish them in the areas most important for mitigating biodiversity loss. Habitat of species at risk often coincides with areas of high human population density or land use [7,8], potentially generating mismatches between economic and conservation priorities. Combined with a lack of political will or funding [9], this can result in imperiled taxa being poorly represented within protected area networks [10]. Given these challenges, optimizing which species-at-risk habitat is proposed for protection is crucial. A promising prioritization strategy is to identify ‘hotspots’ with particularly high taxonomic richness.

There are, however, two important considerations in using the hotspot approach to guide conservation. First, it is unclear how well hotspots of species richness overlap among taxa. To date, hotspots have largely been identified for specific taxonomic groups [11–14]. But hotspots of overall or at-risk species diversity can occur in different places for different groups. For instance, there is limited overlap in hotspots of vertebrates and plants in China [15], and of birds, mammals and invertebrates in the UK [16]. Poor hotspot overlap among taxa complicates prioritizing areas for conservation.

Second, even when hotspots align among taxa, prioritizing hotspots of at-risk species may be controversial when species are rare (and therefore deemed at-risk) in one jurisdiction but widespread elsewhere. Many jurisdictions prioritize taxa for conservation based primarily on the size, range and stability of populations within their borders. Thus, species that only pop into a jurisdiction at the edge of their geographic range (henceforth ‘peripheral’ taxa [17]) can be rare and deemed at risk in that jurisdiction even if they are widespread outside it. This can focus regional conservation efforts on range-edge populations of species that are globally secure, particularly in high-latitude countries that often contain the poleward range-edge of species more common towards the equator [18]. For instance, more than 75% of Canada's nationally at-risk plants and Finland's rare beetles are largely distributed outside these countries [8,19], and most US state lists of threatened birds are dominated by taxa with low global risk [20]. Protecting locally imperilled species has therefore been accused of being ‘parochial’ and coming at the cost of protecting endemic or globally imperilled species [21,22]. However, whether such a trade-off really exists is often unknown. Hotspots of locally at-risk species can co-occur with hotspots of overall diversity [11] and the extent to which hotspots of locally at-risk and globally at-risk species co-occur has rarely if ever been formally assessed.

An excellent case study for exploring how well hotspots of at-risk taxa co-occur, particularly between nationally and globally at-risk species, is Canada. As the world's second largest country and the northernmost country in the Americas, habitat protection in Canada is critical for the species already there and the many predicted to shift northward under climate change. Many nationally at-risk species are peripheral (i.e. only occur in Canada at the northern edge of their range, though this has only been quantified for plants and mammals), thus critics have argued that prioritizing nationally at-risk species for conservation detracts from conserving globally at-risk species [23,24]. However, the geographical overlap between nationally and globally at-risk species has not been quantified. Further, while we know that Canada's nationally at-risk taxa cluster in southern Canada [18,25], it is unclear whether this is driven by species-rich taxonomic groups (e.g. plants, which cluster south [8]), and how well overall hotspots of at-risk species would reflect hotspots of less species-rich groups (e.g. mammals, which have northwestern clusters [11]). Identifying conservation hotspots in Canada is also particularly timely. Habitat loss threatens more than three-quarters of Canada's nationally at-risk species [26], many of which have very little range overlap with protected areas [8,27], and Canada has recently committed to protecting 30% of its land by 2030, up from the 12.5% conserved in 2020 [28,29].

Using range maps of Canada's nationally and globally at-risk taxa (terrestrial taxa + freshwater molluscs), we run three sets of analyses. (i) *Hotspots*. We identify where species-at-risk are concentrated in Canada, and ask (Q1): how well do hotspots overlap among taxonomic groups, and between nationally and globally at-risk species? We expected most hotspots of nationally at-risk species to be in southern Canada, where Canada's biodiversity, human population and land conversion all cluster [25,27], but did not have specific predictions for Q1, given the differing distributions of nationally at-risk plants versus mammals [8,11] and general sense that Canada's globally at-risk species are often in the far north. (ii) *Peripherality.* We quantify the proportion of each at-risk taxon's range in Canada, and ask (Q2): does peripherality (the extent to which taxa only occur in Canada at their range edge) vary among threat statuses and taxonomic groups, and between nationally and globally at-risk species? We expected that nationally at-risk species would be mostly peripheral, and significantly more peripheral than globally at-risk species since national conservation assessment focuses on Canadian range area whereas the IUCN assesses global range area. (iii) *Habitat protection*. (Q3) How well is Canada protecting habitat of species at risk? As many nationally at-risk species occur in highly human-modified southern Canada [30], we were not optimistic about overlap with protected areas.

## 2. Methods

Eligibility for protection under Canada's Species at Risk Act (SARA) is determined by the Committee on the Status of Endangered Wildlife in Canada (COSEWIC). COSEWIC has 10 subcommittees each dedicated to assessing a taxonomic group using quantitative criteria established by the IUCN. Taxa can be species, subspecies or populations. Here we consider terrestrial or mostly terrestrial taxa (excluding freshwater fish and marine species) that COSEWIC has assessed as nationally ‘at-risk’, i.e. threat status of Special Concern (may become threatened or endangered); Threatened (likely to become endangered if threats are not mitigated) or Endangered (facing imminent extirpation or extinction). We ignore taxa deemed 'Extirpated', 'Extinct', 'Data deficient' (i.e. no current Canadian range or reliable range map) or 'Not at risk'.

There are three big-picture steps to taxa being listed as at-risk under SARA. (i) *Prioritization*; COSEWIC subcommittees prioritize which taxa to assess. At this stage COSEWIC prioritizes species (versus lower taxonomic levels) who: have a greater proportion of their range or numbers in Canada or are contracting their range towards Canada; have disjunct or unique populations in Canada; or are most likely to become globally extinct due to greater threats or recent declines [31]. Thus, while not every potentially at-risk species has been assessed yet, COSEWIC assessments should reflect the most at-risk taxa within each taxonomic group. That said, while not an explicit criterion, COSEWIC subcommittees avoid prioritizing species that lack sufficient information to assign a threat status. (ii) *Assessment*; for prioritized taxa COSEWIC commissions, reviews and approves an assessment report based on the ‘best biological information’ available [32]. COSEWIC subcommittees use this report to determine whether the taxon meets IUCN-based criteria for being at-risk, which focus on evidence of recent declines in population size or extent [33]. Assessment initially considers only Canadian populations, then reviews whether adjustments are warranted given the likelihood of demographic rescue from outside Canada [34]. COSEWIC subcommittee recommendations are then reviewed by a cross-taxonomic panel, promoting consistency among taxonomic subcommittees, who makes a final recommendation to the federal government. (iii) *Listing under the Species at Risk Act.* The federal government decides whether to list taxa under SARA after considering the socioeconomic implications of COSEWIC's recommendation [32]; we use COSEWIC assessments rather than SARA listings as they more closely reflect biology.

### (a) Data collection

#### (i) COSEWIC range maps

COSEWIC assessment reports are available as PDFs and generally include Canadian and global range maps of the taxon [35]. For all taxa except birds (see §2a(ii)), we updated a database of digitized COSEWIC range maps [8]. Thirty-four taxa had global range maps that were not properly digitizable (maps incomplete, imprecise or in a projection that could not be digitized), but Canadian range maps that were. For these taxa, we digitized Canadian ranges only. These taxa were excluded from peripherality analyses (Q2) but included in the hotspot and protected area analyses (Q1, Q3) for which only Canadian ranges were needed (table 1). Full details on digitization are in the electronic supplementary material.

**Table 1.** Sample sizes for terrestrial or mostly terrestrial taxa deemed at-risk in Canada. Nationally at-risk taxa (A to D) are species, subspecies or populations with a COSEWIC status of Special Concern, Threatened or Endangered. Globally at-risk species (E to G) are species with an IUCN status of Vulnerable, Endangered or Critically Endangered. Reading from A to D and from E to G indicates how many taxa had to be excluded from our analyses due to the lack of adequate range maps. Digitized range maps for nationally at-risk taxa (C and D) were derived from COSEWIC assessments for all taxa except birds; bird range maps were obtained from BirdLife International. Numbers in columns C, D and G indicate the final sample sizes for Questions 1, 2 and 3. For globally at-risk species without an IUCN range polygon, we used Canadian range maps from COSEWIC when available to identify hotspots (Q1) and overlap with protected areas (Q3) in Canada, hence numbers in G are sometimes greater than in F.

	nationally at-risk taxa				globally at-risk species		
	A. populations with a COSEWIC status	B. taxa in A with a COSEWIC range map^a^	C. taxa with a digitized Canadian range map (Q1, Q3^b^)	D. taxa in C with a digitized global range map (Q2)^c^	E. taxa in Canada on IUCN Red List	F. taxa in E with an IUCN range polygon	G. taxa in E with a digitized range map (Q1, Q2, Q3)
birds	76	NA	56	54	10	10	10
mammals	42	41	37	36	7	7	7
reptiles	41	34	32	26	3	1	3
amphibians	27	24	21	17	0	0	0
arthropods	70	66	60	54	12	6	9
molluscs	40	39	34	32	17	14	15
vascular plants	207	200	184	171	21	15	17
mosses	20	20	17	10	0	0	0
lichens	23	23	21	14	6	2	3
total	546	NA	462	414	76	55	64

^a^If populations in A were from the same species and only one range map was provided for that species, they were merged in B.

^b^Q3: analyses of hotspot overlap with protected areas uses *n* in C; analyses of range overlap with protected areas by threat status has slightly lower *n*, as populations of the same species with different threat statuses had to be excluded.

^c^If the populations merged in B did not have the same COSEWIC status, they were excluded from D.

#### (ii) BirdLife range maps

Most at-risk birds in Canada are migratory, so their range maps had to be divided between resident, breeding, non-breeding and passage (e.g. migratory stopovers) ranges to meaningfully compare their Canadian and global distributions. Seasonal ranges are not always separated in COSEWIC reports, so we obtained polygon range maps with this information from BirdLife International [36]. BirdLife maps are equivalent to IUCN maps for other vertebrates, and previous work found that IUCN and COSEWIC maps were generally comparable in their level of detail [11]. From BirdLife maps, we retained only ranges that overlapped a polygon shapefile of Canada. When COSEWIC assessed a bird subspecies or population, we used the BirdLife map if it could be separated by subspecies or population, otherwise excluded the taxon (table 1). For species that spend multiple seasons in Canada (e.g. both breeding and passage), we merged the seasonal polygons and used this merged shapefile for analyses. Thus, we compared each species's Canadian range with their corresponding seasonal global range. For instance, if a species only uses land in Canada during its breeding and passage season, we compared its Canadian breeding + passage range to its global breeding + passage range.

#### (iii) IUCN range maps

To map globally at-risk species in Canada, we searched the IUCN Red List of Threatened Species [37] for species in the categories Vulnerable, Endangered or Critically Endangered (i.e. ‘at-risk’), whose ‘land region’ included Canada and assessment scope = ‘global’. We downloaded the polygon shapefile of each species's range from IUCN, excluding nine species whose IUCN reports were flagged as needing updating and nine species whose Canadian ranges were defined as ‘vagrant’ and did not appear in their global range maps. We removed polygon areas defined by IUCN as ‘possibly extinct’. For species without an IUCN range polygon, we used the species's global range polygon digitized from its COSEWIC report if available (nine non-bird species; table 1).

### (b) Data extraction

Data extraction and analyses were done in R (v.4.1.0, [38]). To identify hotspots with unusually high concentrations of at-risk taxa, we reprojected each taxon's digitized range map (see electronic supplementary material) and overlaid these on a map of Canada. We then superimposed a 100 × 100 km grid, dividing Canada into 1276 grid cells, and counted the at-risk species in each cell. We chose 100 × 100 km as it produced enough cells to reveal interesting patterns, without overestimating the accuracy of range maps, whose borders can vary depending on how polygons are drawn [39]. For each of the nine taxonomic groups of nationally at-risk taxa, nationally at-risk taxa overall and globally at-risk species overall, we identified which cells were hotspots. As per previous studies [11,16,40], we defined hotspots as the cells with the highest species richness up to a maximum of 5% of the 1276 grid cells (max. 63 cells). For example, for mammals, there were 55 cells that included at least six at-risk taxa, and 118 cells that included five taxa. Cells with five mammal taxa were therefore not counted as hotspots, as doing so would have resulted in more than 63 hotspot cells. Thus, the number of hotspots varied from 42 to 62 hotspot cells per taxonomic group.

For Question 2 (*Peripherality*), we reprojected maps (electronic supplementary material), then quantified the proportion of each taxon's Western Hemisphere range that occurs in Canada. This is the inverse of peripherality; the smaller the proportion is, the more peripheral the taxon is (i.e. the less of its range occurs) in Canada. We deemed Western Hemisphere range to be a more biologically relevant denominator than global range, since the difficulty of moving between hemispheres makes it unlikely that populations on other continents would substantially impact Canadian populations.

For Question 3 (*Habitat protection*), we calculated the proportion of each taxon's Canadian range and of each hotspot that overlapped government-protected areas. We used the Canadian Protected and Conserved Areas Database [28], which includes polygon shapefiles of ‘terrestrial protected areas' and ‘terrestrial areas with other effective area-based conservation measures’ protected federally, provincially or municipally. We intersected protected area shapefiles with (i) each at-risk taxon's range polygon to calculate the km^2^ of protected habitat within each Canadian range; and (ii) the map of Canada divided into 100 × 100 km grid cells from Q1 to calculate the km^2^ of protected habitat in each cell. To calculate the proportion of protected habitat per cell, we divided the area of protected habitat by the cell's total land area in Canada (10 000 km^2^ or less if grid cell included ocean or land in the USA).

### (c) Data analyses

Analyses assessed differences among taxonomic groups for nationally at-risk species and compared nationally and globally at-risk species. We did not split analyses of globally at-risk species by taxonomic group, due to small sample sizes in several groups (table 1). See ‘Data accessibility’ for link to archived data and code.

#### (i) Hotspot analyses

To assess hotspot overlap among pairs of groups (e.g. birds and mosses, nationally at-risk and globally at-risk taxa), we counted the cells that were hotspots for both groups, then calculated the percentage of shared hotspots. As different taxonomic groups had different numbers of hotspots (see §2b), we calculated overlap twice, one with each group as a denominator, then used their average. For example, birds (48 hotspots) and mosses (42 hotspots) shared 6 hotspot cells, resulting in a mean percentage overlap of 13% (i.e. the mean of 6/48 × 100 and 6/42 × 100).

#### (ii) Peripherality analyses

We tested whether the proportion of a taxon's Western Hemisphere range in Canada (proportional response) varied among taxonomic groups and threat statuses using generalized linear models (GLMs). Our response variables for Q2 (and Q3) are proportions that do not derive from a binomial process, so all models use a beta-regression error structure (*glmmTMB* package version 1.1.2 [41]; full details of beta-regression in electronic supplementary material).

First, we tested whether ‘range proportion in Canada’ differed among COSEWIC statuses and/or taxonomic groups of nationally at-risk taxa (Model 1: proportion range in Canada∼COSEWIC status×taxonomic group). Each nationally at-risk taxon contributed one data point (sample sizes in table 1D), consistent with taxon-based conservation policy that treats taxa as independent units (e.g. Red lists). We grouped vascular plants and mosses together into taxonomic group = ‘plants’ due to the small number of at-risk mosses (table 1). We tested the significance of fixed effects using likelihood ratio tests, which compare models with and without the fixed effect, compared to a *χ*^2^ distribution. As the two-way interaction was not significant ($\chi_{\mathrm{df}14}^{2}=16.8$, *p* = 0.26), we dropped it from the final model to facilitate interpretation of main effects. For significant main effects, we used least-squared mean contrasts to determine which taxonomic groups or threat statuses differed from each other (*lsmeans* package version 2.30.0; [42]).

Second, we tested the widely held assumption that nationally at-risk taxa are more peripheral in Canada than globally at-risk species. We ran two models, each with one fixed effect ‘threat status’. In Model 2, we tested whether peripherality differed among the different levels of endangerment defined by COSEWIC and IUCN. These bodies use different status definitions and consider different geographic extents (Canada versus global), so Model 2 had six categories of ‘threat status’; the three at-risk statuses from COSEWIC and the three from IUCN. In *Model 3,* we collapsed these into two categories: ‘nationally at-risk’ or ‘globally at-risk’. In both models, each nationally at-risk taxon and each globally at-risk species contributed one data point (sample sizes in table 1D,G). Because 42 taxa are considered at-risk by both COSEWIC and IUCN, and therefore occur in the data twice, we used generalized linear mixed models with a random intercept for ‘species’ (proportion range in Canada∼threat status + (1| species)). Significance of ‘threat status’ and differences among statuses were determined as in Model 1.

#### (iii) Protected areas analyses

We assessed how well hotspots of at-risk taxa were protected compared to less species-rich cells. Spatial data with grid cells as the unit of observation are notoriously spatially auto-correlated, potentially overestimating statistical power. We therefore present descriptive patterns rather than statistical comparisons. First, we looked at the proportion of each grid cell protected, comparing between hotspot cells and non-hotspot cells for nationally at-risk and globally at-risk hotspots. Second, we explored whether the proportion of a cell that was protected varied with the number of at-risk taxa in the grid cell, again for hotspot versus non-hotspot cells for nationally at-risk versus globally at-risk taxa.

We next tested how well ranges of at-risk taxa were protected and whether this varied with threat status, using taxa as the unit of observation as in Models 1 to 3. We ran one GLM for nationally at-risk taxa (*Model 4:* proportion Canadian range protected∼COSEWIC status) and one for globally at-risk species (*Model 5:* proportion Canadian range protected∼IUCN status).

If many nationally at-risk taxa have little of their Canadian range protected (e.g. [8]), we interpret this as government failure to prioritize species-at-risk habitat for protection. However, an alternate explanation is that species outside protected areas are simply more likely to be deemed at-risk by COSEWIC. We think this is unlikely, as habitat protection is not explicitly considered in prioritization or assessment, but threats can be considered during prioritization and species in protected areas are hopefully less likely to experience population and range declines. To gain insight, we tested whether habitat protection varied with time since taxa were deemed at-risk. If governments are failing to protect habitat of species at risk, we expect no relationship between habitat protection and time since assessment. If governments are duly protecting habitat of species at risk but taxa with little habitat protection are more likely to be assessed as at-risk, we expect the most recently assessed taxa to have the least of their range protected (see electronic supplementary material).

## 3. Results

### (a) Hotspots of at-risk taxa

**Figure 1.**
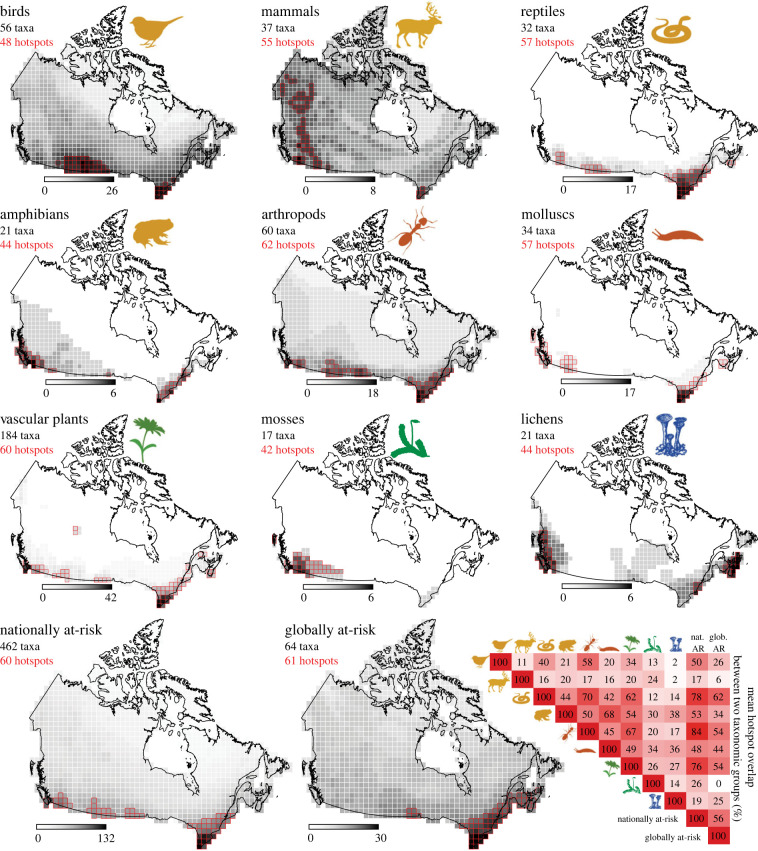
Hotspots of at-risk taxa in Canada. Maps are overlaid with a 100 × 100 km grid, which divides Canada into 1276 cells. In each map, cells are shaded according to the number of at-risk taxa they contain, such that black always indicates the maximum hotspot diversity for that group (greyscale legends show the maximum number of at-risk taxa per grid cell for each map). Red outlines show hotspot cells, i.e. the cells with the most at-risk taxa up to a maximum of 5% of the total grid cells. Top three rows show nationally at-risk taxa separated by taxonomic group (yellow icons = vertebrates; orange = invertebrates; green = plants; blue = lichens). Bottom two maps show all nationally at-risk taxa (left) and globally at-risk species found in Canada (right) across taxonomic groups. Matrix of overlap (bottom right) indicates the percentage of hotspot cells that coincide for each pairwise combination; darker background colour indicates greater hotspot overlap.

In Canada, hotspots of nationally at-risk taxa generally clustered along the southern border (figure 1). This was true for most taxonomic groups, with the notable exceptions of at-risk mammals, whose diversity hotspots followed the western mountains, and at-risk lichens, whose hotspots were mostly coastal. When all of Canada's nationally at-risk taxa in our data were combined (figure 1, bottom left map), their hotspots clustered along Canada's southern border from western Canada and the prairies to the Great Lakes and St Lawrence River regions.

Hotspots showed substantial taxonomic overlap. Taxonomic groups shared 32% of their hotspot cells on average (figure 1). Overlap between hotspots of individual taxonomic groups and hotspots of nationally at-risk taxa overall was even higher (mean = 50.1%). More than 75% of the hotspots of at-risk reptiles, arthropods and vascular plants overlapped with nationally at-risk hotspots (figure 1). Hotspots of nationally at-risk taxa contained as many as 132 at-risk species, and 40% contained at-risk species from all nine taxonomic groups. Together, the 4.4% of land in Canada included in nationally at-risk hotspots was home to 80% of the 462 nationally at-risk taxa in our data, including: 97% of the reptile taxa, 95% of amphibians, 92% of arthropods, 89% of birds, 85% of molluscs, 76% of vascular plants, 65% of mosses, 65% of mammals and 62% of lichens. While groups with the most at-risk species tended to have high overlap with national hotspots, overlap was not solely driven by richness. For example, there were three times as many at-risk vascular plant taxa in our data (184) than the next highest group (arthropods, 60 taxa), but arthropod hotspots had higher overlap with nationally at-risk hotspots (figure 1).

Globally at-risk species in Canada showed a remarkably similar distribution to nationally at-risk taxa (figure 1, bottom maps). Contrary to our expectation that globally at-risk species might be distributed farther north, hotspots of globally at-risk species also clustered along Canada's southern border, but were almost entirely in eastern Canada from the Great Lakes to the southern Maritimes. More than half (56%) of globally at-risk hotspots were also hotspots of nationally at-risk taxa. Together, these 34 overlapping hotspots, representing only 2.4% of Canada's land area, were home to 67% of the globally at-risk taxa and 43% of the nationally at-risk taxa in our data.

### (b) Peripherality of at-risk taxa in Canada

**Figure 2.**
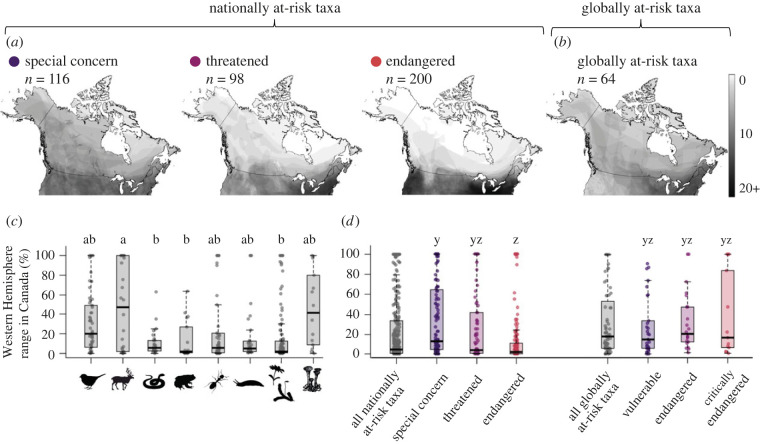
Geographical distribution and peripherality of at-risk taxa in Canada. Maps show Canadian ranges of (*a*) nationally at-risk taxa, separated by their COSEWIC threat status, and (*b*) globally at-risk taxa, combined across IUCN at-risk threat statuses. Grey range polygons are semi-transparent, so areas with more overlapping polygons appear darker (all maps use the same grey scale). Coloured dots correspond to threat status, from least (purple) to most (red) imperilled. (*c*) Nationally at-risk taxonomic groups differed in how peripheral they were; bars without shared letters are significantly different (Model 1). (*d*) Nationally at-risk taxa were not significantly more peripheral in Canada than globally at-risk taxa when compared among the six threat statuses (Model 2; coloured bars) or between nationally and globally at-risk taxa overall (Model 3; grey bars). Box plots show the median (thick middle line), 25th and 75th percentiles (box), 1.5 × the interquartile range (whiskers) and raw data (horizontally jittered points). Results are for the 414 nationally at-risk taxa and 64 globally at-risk taxa that had digitizable Canadian and global range maps (table 1).

Most nationally at-risk taxa only occur in Canada at the northern edge of their range (figure 2*a*). While most taxonomic groups contained some endemic taxa (47 taxa had 100% of their Western Hemisphere range in Canada, e.g. Vancouver Island marmot, Banff Springs snail and Magdalen Islands grasshopper), 71% of nationally at-risk taxa had less than 20% of their Western Hemisphere range in Canada (mean = 23.8% of range in Canada, median = 4.4%). The extent to which taxa were peripheral in Canada varied among COSEWIC threat statuses ($\chi_{\mathrm{df}2}^{2}=15.1$, *p* < 0.001) and taxonomic groups ($\chi_{\mathrm{df}7}^{2}=21.7$, *p* = 0.003; Model 1). At-risk mammals had significantly more of their range in Canada (i.e. were less peripheral) than at-risk reptiles, amphibians or plants (figure 2*c*). The most imperilled taxa had significantly less of their range in Canada (i.e. were more peripheral) than the least imperilled at-risk taxa (figure 2*d*).

Contrary to our predictions, many globally at-risk species also occurred in Canada only at their northern range edge. Fifty-three per cent of globally at-risk species in Canada had less than 20% of their Western Hemisphere range in Canada (mean = 31.3% of range in Canada, median = 17.8%). All IUCN and COSEWIC threat categories contained both taxa with less than 5% of their range in Canada, and taxa that are endemic or almost endemic (IUCN Vulnerable taxa had up to 90.7% (for polar bear) of their range in Canada). The extent to which taxa were peripheral in Canada varied significantly among the six threat statuses (Model 2: $\chi_{\mathrm{df}5}^{2}=21.1$, *p* < 0.001; figure 2*d*, coloured bars), but only because nationally Endangered taxa had less of their range in Canada than Special Concern taxa, as found in *Model 1*. No category of nationally at-risk taxa was more peripheral than any category of globally at-risk species, nor did peripherality differ between nationally and globally at-risk taxa overall (Model 3; $\chi_{\mathrm{df}1}^{2}=2.8$, *p* = 0.09; figure 2*d*, grey bars).

### (c) Habitat protection of at-risk taxa

**Figure 3.**
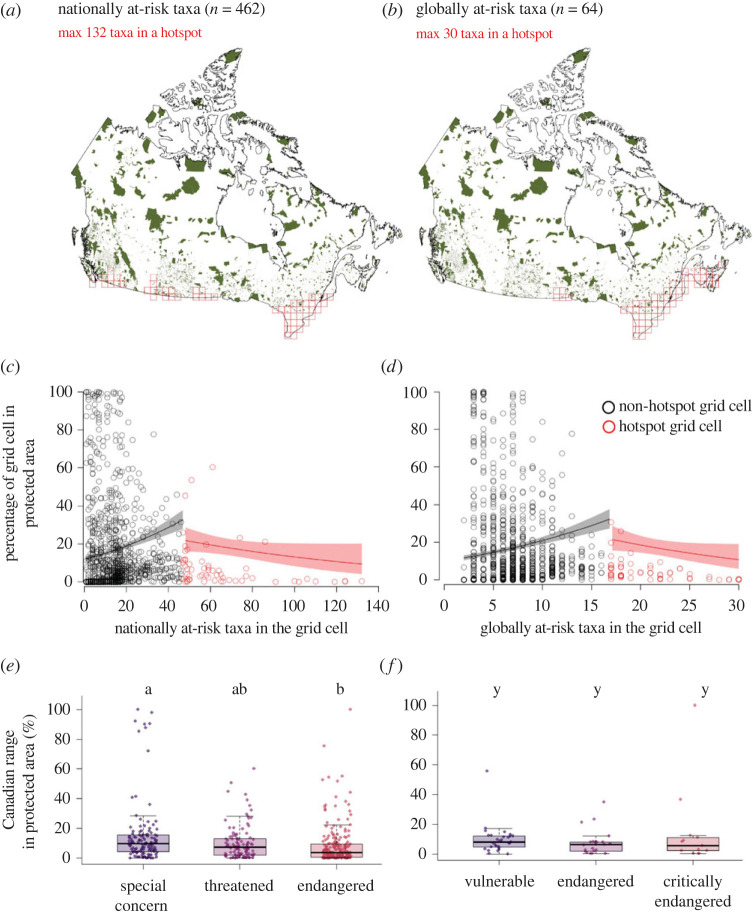
Habitat protectionof nationally at-risk taxa (left) and globally at-risk taxa (right) in Canada. (*a*,*b*) location of Canada's publicly protected and conserved areas (green), and hotspots of at-risk taxa (from figure 1) outlined in red. (*c*,*d*)Protection tends to increase with the number of at-risk taxa in non-hotspot cells (black), but not for cells that are hotspots of at-risk taxa (red). Each point represents one of the 1276 grid cells in Canada. Trends lines and shading for visualization were extracted from beta-GLMs; since these do not account for potential spatial autocorrelation, true 95% CI are likely to be wider. (*e*,*f*) Percentage of at-risk taxa's Canadian range included in protected areas. Differing letters represent significant differences among (*e*) COSEWIC threat statuses (Model 4) or (*f*) IUCN threat statuses (Model 5). Sample sizes are in table 1. Box plot formats are as in figure 2.

Areas with particularly high concentrations of at-risk species (hotspots) are not particularly well protected. Whereas hotspots of nationally and globally at-risk taxa cluster in southern Canada, Canada's largest expanses of protected habitat are further north (figure 3). Hotspots of nationally at-risk taxa were on average 8.0% protected (median = 3.5% of cell protected), whereas cells that were not hotspots were 12.9% protected (median = 2.4%). Cells that were hotspots of globally at-risk taxa had even less protection, with an average of 4.7% of the grid cell protected (median = 2.7%). For both nationally and globally at-risk taxa, the percentage of a grid cell protected tended to increase as the number of at-risk taxa in the cell increased, but only for non-hotspot cells; figure 3*c,d*). Of the 1276 grid cells across Canada, 34% contained no protected areas, while 0.5% were fully protected. Only 6.8% of the land in nationally at-risk hotspots was protected. Protection is even lower for land in hotspots of globally at-risk taxa (5.1% protected), and lower still for areas that were hotspots for both nationally and globally at-risk taxa (3.8% protected).

The Canadian ranges of individual at-risk taxa were also poorly protected on average. Nationally and globally at-risk taxa had only 6.2% and 7.5% (median) of their Canadian range protected, respectively. Most nationally at-risk taxa (67%) and globally at-risk species (70%) had less than 10% of their Canadian range protected. This is despite the fact that many at-risk taxa have small range areas in Canada (median area in Canada: nationally at-risk taxa = 12 157 km^2^, globally at-risk taxa = 112 113 km^2^). Sixteen nationally at-risk taxa had no protected areas in their range polygon at all. Habitat protection differed among COSEWIC statuses, with the most imperilled taxa having *less* of their Canadian range protected than the least imperilled at-risk taxa (Model 4: $\chi_{\mathrm{df}2}^{2}=17.4$, *p* < 0.001; figure 3*e*). Habitat protection did not vary with the time since taxa were deemed at risk (electronic supplementary material, figure S1); in other words, there is no evidence that being deemed nationally at-risk leads to subsequent habitat protection. Habitat protection was equally low among globally threatened species and did not differ among IUCN statuses (Model 5: $\chi_{df2}^{2}=0.62$, *p* = 0.73; figure 3*f*).

## 4. Discussion

Our analyses of imperilled taxa in Canada support some preconceptions about their spatial distribution, but counter others. First, our results confirm that most species considered at-risk in Canada only occur in Canada at the northern edge of their range (figure 2). This aligns with previous qualitative assessments [18], but had not been quantified across taxa before. However, our results do not support the perception that nationally at-risk taxa are more peripheral than globally at-risk species in Canada. While globally at-risk species did have slightly more of their range in Canada on average, they also clustered towards the southern border and 53% had less than 20% of their range in Canada (figure 2). These results underscore that conservation of at-risk species and biodiversity in Canada, as in many high-latitude countries [18,19,43], is fundamentally linked to the conservation of range-edge populations.

Our results also challenge the perception of an inherent trade-off in protecting nationally at-risk versus globally at-risk species. As so many of Canada's nationally at-risk taxa only occur in Canada at the northern edge of their range, there has been significant debate about how to prioritize their conservation [23,44] and sustained criticisms that doing so comes at the cost of protecting globally imperilled species [24,45]. Our results suggest that at least in terms of habitat protection, this is a false dichotomy. More than half (56%) of the areas with the highest concentration of nationally at-risk taxa in Canada were also hotspots of globally at-risk species in Canada (figure 1). Hotspots of nationally at-risk taxa encompassed 67% of the globally at-risk taxa in our data, showing that protecting one threatened group can significantly benefit the other.

Not only do hotspots of nationally at-risk taxa contain many globally at-risk taxa, they are also taxonomically diverse. A single nationally at-risk hotspot contained up to 132 at-risk taxa (figure 1), and all contained at-risk species from at least six taxonomic groups. However, nationally at-risk hotspots did not represent at-risk mammals as well as they did other taxa. This is important as non-human mammals often garner disproportionate conservation interest from human mammals [46–48]. Hotspots of nationally at-risk mammals do contain high overall mammal diversity [11], so conservation efforts in these areas are certainly worthwhile. However, our results caution that using at-risk mammals as charismatic flagship species [49] will not necessarily focus conservation efforts towards the habitat most needed by other at-risk taxa. Protecting habitat in nationally at-risk hotspots, on the other hand, can have a significant conservation benefit for at-risk mammals. Hotspots of nationally at-risk species together contained more than 50% of the at-risk taxa in each taxonomic group, which is highly promising for planning diverse protected areas.

Any work on species at-risk reflects both biology (location and demography of populations) and policy (how statuses are determined). It is worth exploring how policy decisions might affect interpretation of our results. First, COSEWIC has limited funds for assessments so many subcommittees have wait lists, particularly subcommittees whose taxonomic groups are more diverse (e.g. vascular plants). Thus, the true distribution of nationally at-risk hotspots may more strongly reflect the centres of diversity of these groups. For example, if at-risk but unassessed plants are distributed similarly to assessed and at-risk plants (figure 1), nationally at-risk hotspots would cluster even more strongly in southern Canada.

Second, it has been suggested that taxonomic convergence of hotspots could be an artefact of data availability. COSEWIC generally only commissions reports if there is likely sufficient data to determine a threat status, and the required data are more available for some taxa and some places than others. Could apparent southern hotspots of less well-known taxonomic groups (mosses, lichens, molluscs, arthropods) simply reflect where taxonomic experts live and work, thus artificially creating hotspots in southern Canada? We posed this question to these COSEWIC subcommittees. Most felt that the hotspots in figure 1 reflect the true distribution of species richness, particularly of small-ranged species, rather than lack of information on northern species. Further, amphibians, reptiles and birds are all well-studied and still have hotspots clustered in southern Canada. Could apparent taxonomic overlap result from cross-taxonomic studies of at-risk species (e.g. if arthropods are more likely to be studied, and therefore to have sufficient data for COSEWIC assessment, if they interact with at-risk plants)? While possible, this would require that cross-taxonomic studies frequently detect rare taxa and are common enough to yield sufficient data for assessment. Rather, fieldwork commissioned by COSEWIC is species-specific so would not yield useable data on other taxa, and cross-taxa academic studies appear rare (only 2 of 41 papers on Canadian populations of at-risk plants included data on other groups [8]). Thus, while there may well be rare northern species that have not been assessed, cross-taxonomic hotspots in southern Canada do not seem to be artefacts of data availability.

Strong overlap in hotspots of different taxonomic groups and threat levels should facilitate habitat protection to promote species-at-risk recovery, but we see little evidence that protected areas have been planned with species at risk in mind. The amount of land protected increased with the number of at-risk species in non-hotspots cells (figure 3), which is an improvement over a complete lack of relationship 15 years ago [50]. However, this effect was slight and did not hold for hotspots of at-risk taxa. Nor did we find evidence that new protected areas were being selected with species at risk in mind (electronic supplementary material), and most nationally and globally at-risk species have only a tiny fraction of their Canadian range protected (figure 3). Areas that are hotspots for both nationally at-risk and globally at-risk taxa should be of the highest conservation priority, but only 3.8% of these areas are publicly protected.

Granted, habitat protection is not the only way to help at-risk species, and governmental protection is not the only way to protect habitat. Privately protected and indigenous-governed lands comprise critically important habitat for species at risk in Canada and globally, and reversing biodiversity declines requires broad cooperation and diverse approaches [51–53]. Still, given government commitments to help at-risk species and that habitat loss threatens most at-risk species [26], public protection of habitat is also critical. While linking protected area designation to species-at-risk habitat is recommended in extra-governmental frameworks (e.g. Key Biodiversity Areas [54]), our results suggest there has been a disconnect between designation of species-at-risk and designation of publicly protected areas.

Why is Canada not doing better at protecting species at risk within its borders? The disconnect could reflect delays and biases in identifying critical habitat or implementing recovery plans [55–57], or lack of coordination between the disparate government departments responsible for species-at-risk recovery planning and protected areas. It strongly suggests that protected area selection has so far been driven by objectives other than protecting species at risk. While protected area objectives can include historical, scenic or cultural significance [58], designation may also be influenced by minimizing conflict with industry and developers, rather than optimizing species-at-risk conservation [59,60] as in many protected area networks worldwide [10,61]. Finally, while most federal-listed species occur in southern Canada, most federally governed land occurs in northern Canada. Thus, habitat protection in Canada's biodiversity hotspots requires cooperation across political levels, which is not always forthcoming [62].

The difficulty of protecting habitat for species at risk underscores the importance of identifying priority areas that can promote as many conservation objectives as possible. Our findings of high spatial overlap among at-risk taxa are great news: it means Canada's recent commitment to protect more natural area could easily be used to promote species-at-risk recovery. Further, protecting natural landscapes in densely populated areas like southern Canada, where both nationally and globally at-risk species cluster, can benefit the many people living there [63] who are increasingly seeking natural areas [64,65].

We realize that habitat protection generally happens at a smaller scale that the 100 × 100 km grid cells used in our hotspot analysis. The scale of our grid cells makes analytical sense for a country as large as Canada, but could obscure nuance in protected areas. As most species occupy patchily distributed habitat, species rarely occupy an entire hotspot grid cell or all the area within their range polygons [66]. Thus, the actual proportion of a species's Canadian range protected could be higher than suggested by our data if existing populations are found disproportionately in protected areas. However, protected areas are also small and patchy, thus our estimates of habitat protection could be too high for some species, if protected areas in their range polygon do not actually include existing populations. Future protected areas are likely to be much smaller than our grid cells, especially in southern Canada where private land ownership is high, and might not encompass a hotspot's full suite of overlapping taxa. Even so, coarsely mapping Canada's nationally and globally at-risk hotspots provides a useful starting point for future, more local-scale analyses to pinpoint priority areas. Further, patchy species distributions mean that small parks and protected areas could have a disproportionate benefit for individual species at risk.

Another reason to protect habitat in hotspots of species at risk, even if not all species in the region occur in the local protected area, is that range shifts driven by climate change are already changing taxonomic overlap [67] and habitat use of at-risk species [68]. Protecting land within the taxonomically diverse, at-risk hotspots we identified could offer habitat refuges to surrounding species whose ranges do not yet overlap that area [69]. Northward range shifts are especially important given our finding that 71% and 53% of the nationally and globally at-risk taxa in Canada occur at their northern range edge. Protecting habitat in at-risk hotspots would not only protect a taxonomically diverse set of at-risk populations, but could also provide essential corridors for edge populations to shift towards higher latitudes and track a changing climate [18,70]. Protecting habitat within Canada's at-risk hotspots is therefore crucial for Canada's current and future biodiversity.

## Data Availability

Data used in analyses are archived on Borealis, the Canadian Dataverse Repository: https://borealisdata.ca/. Supplementary method details and analyses are provided in the electronic supplementary material [71].
